# The Impact of Dual-Tasks and Disease Severity on Posture, Gait, and Functional Mobility among People Living with Dementia in Residential Care Facilities: A Pilot Study

**DOI:** 10.3390/s24092691

**Published:** 2024-04-24

**Authors:** Deborah A Jehu, Ryan Langston, Richard Sams, Lufei Young, Mark Hamrick, Haidong Zhu, Yanbin Dong

**Affiliations:** 1 Department of Community & Behavioral Health Sciences, Institute of Public and Preventative Health, Augusta University, Augusta, GA 30912, USA; 2 Georgia War Veterans Nursing Home, Augusta, GA 30901, USA; risams@augusta.edu; 3 School of Nursing, University of North Carolina, Charlotte, NC 28081, USA; lyoung57@charlotte.edu; 4 Department of Cellular Biology and Anatomy, Medical College of Georgia, Augusta University, Augusta, GA 30912, USA; mhamrick@augusta.edu; 5 Georgia Prevention Institute, Medical College of Georgia, Augusta University, Augusta, GA 30912, USA

**Keywords:** Alzheimer’s disease, multi-task, balance, cognition, cognitive–motor interference, dual-task interference, nursing home, assisted living

## Abstract

Gait speed and timed-up-and-go (TUG) predict cognitive decline, falls, and mortality. Dual-tasks may be useful in cognitive screening among people living with dementia (PWD), but more evidence is needed. This cross-sectional study aimed to compare single- and dual-task performance and determine the influence of dementia severity on dual-task performance and interference. Thirty PWD in two residential care facilities (Age: 81.3 ± 7.1 years; Montreal Cognitive Assessment: 10.4 ± 6.0 points) completed two trials of single- (feet apart) and dual-task posture (feet apart while counting backward), single- (walk 4 m) and dual-task gait (walk 4m while naming words), and single- (timed-up-and-go (TUG)), and dual-task functional mobility (TUG while completing a category task) with APDM inertial sensors. Dual-tasks resulted in greater sway frequency, jerk, and sway area; slower gait speed; greater double limb support; shorter stride length; reduced mid-swing elevation; longer TUG duration; reduced turn angle; and slower turn velocity than single-tasks (*p_s_* < 0.05). Dual-task performance was impacted (reduced double limb support, greater mid-swing elevation), and dual-task interference (greater jerk, faster gait speed) was related to moderate-to-severe compared to mild PWD. Moderate-to-severe PWD had poorer dynamic stability and a reduced ability to appropriately select a cautious gait during dual-tasks than those with mild PWD, indicating the usefulness of dual-tasks for cognitive screening.

## 1. Introduction

Dementia is a significant and growing healthcare issue in the geriatric population [1]. People living with dementia (PWD) experience falls at a rate two times higher than those without dementia [2]. Concurrent cognitive and motor impairments place PWD at increased risk for falls [3], as they have shown greater postural sway, slower walking speed, decreased step length, and larger double limb support than those without dementia [4,5]. These cognitive and motor impairments can make rehabilitation challenging while posing difficulties for residential care facility placement and management [6]. It is estimated that over 50% of PWD have not been formally diagnosed [7,8], in part due to factors such as stigma, a lack of education of dementia signs and symptoms, system resource constraints, and insensitivity to assessments [9]. As a result, the majority of PWD receive their diagnosis after the onset of clinical symptoms [10], thereby narrowing the window of opportunity for effective therapeutic intervention [11]. The Mini-Mental State Examination (MMSE) is one of the most popular screening tools for cognitive impairment [12]. However, like other cognitive assessments, the MMSE is not sensitive to changes in cognitive capabilities, has floor and ceiling effects, is copyrighted, is subject to the effects of language and education, and is unable to detect the progression of dementia [13,14]. There are no cognitive assessments that can be exclusively used to diagnose any mental status disorder, and hence, dementia diagnoses are currently based on clinical judgment of (1) the loss of two or more cognitive abilities (often memory with at least one other domain such as language, visuospatial, and executive), (2) a distinct decline from prior level of cognitive function, and (3) impaired performance in daily activities (e.g., social, occupational, self-care) [10]. Therefore, it is important to identify more accurate methods to screen and monitor cognitive status.

Performing two tasks simultaneously (i.e., dual-tasks), such as walking while performing a cognitive task, may be useful in cognitive screening and monitoring [15,16]. Dual-tasks require reallocation of cognitive resources, therefore making dual-tasks more difficult [17]. Dual-tasks have been sensitive to discriminate between those with and without dementia [15,16,18,19], predict future dementia diagnosis [20], and have exposed subtle impairments that were not obvious under a single-task test condition [21,22,23]. However, no studies have examined whether dual-tasks are sensitive to detect impairments across dementia severities. Dual-tasks have also had higher accuracy in predicting future dementia diagnoses than single-tasks among older adults with mild cognitive impairment [24]. The dual-task timed-up-and-go (TUG) with a category cognitive task has better discriminant validity between those with and without dementia than the single-task TUG [25]. Additionally, certain dual-tasks (e.g., dual-task “Figure 8 walking test”) may be more predictive of falls than single-tasks [26,27]. However, further work is needed to confirm these findings because other dual-tasks, such as gait speed [28] and the TUG [29,30], have shown similar predictability of falls and frailty as single-tasks among older adults. Altogether, a better understanding of the role of dual-tasks during posture and gait may improve cognitive screening and monitoring among PWD.

While dual-tasks can promote automatic postural control (i.e., decreased amplitude and variability of postural sway) by drawing attention away from focusing on posture and onto a secondary cognitive task among healthy young adults [31,32], the increased attention demand of dual-tasks provokes greater conscious control of posture (i.e., reduced automaticity) in cognitively and physically frail aging populations [15,33,34,35]. Impairments in cognition and mobility are likely due to the neurodegeneration of shared brain areas among PWD [17]. Community-dwelling PWD have shown greater sway area and path length during dual- compared to single-task posture, likely, in part, because the control processes associated with dual-tasks (e.g., divided attention, coordination, switching attention) are impaired [15,34]. However, it is unclear how dual-tasks influence other measures of postural control among PWD in residential care facilities or whether dual-task postural control is linked with dementia severity.

Dual-tasks alter spatial and temporal gait parameters relative to single-tasks, such as greater trunk instability [36], slower gait speed [24,37,38], and greater stride time variability [36] in healthy populations. However, this dual-task interference in gait tends to be exacerbated in PWD [36,39,40,41]. The majority of the dual-task gait literature in PWD employed more conventional gait parameters (e.g., gait speed) rather than spatial or other temporal parameters as indicators of dynamic stability [36,41]. Limited research has characterized the impact of dual-tasks on gait parameters in PWD, which have all been in community dwellers [28,39,40,41]. Residential care settings have been linked with a greater risk for falls and cognitive decline compared to living in the community [42]. Despite the potential usefulness of a dual-task assessment to screen for cognitive impairment, to our knowledge, no studies have determined whether dual-task gait or functional mobility can be used as a marker of dementia severity among PWD in residential care facilities.

A better understanding of the influence of dual-tasks on posture, gait, and functional mobility, as well as whether they can be used as markers of dementia severity, may inform cognitive screening procedures among PWD. Additionally, PWD in residential care facilities have less functional independence than those living in the community [43], and functional independence may be an indicator of dual-task posture and gait [40], yet dual-task posture and gait have yet to be investigated among PWD in residential care facilities. Accordingly, the purpose of this study was to examine the impact of dual-tasks on posture, gait, and functional mobility in comparison to single-tasks among PWD in residential care facilities using wearable inertial sensors. Our second aim was to determine whether dementia severity influenced dual-task performance and interference of posture, gait, and functional mobility among PWD in residential care facilities using wearable inertial sensors.

## 2. Materials and Methods

### 2.1. Study Design 

This was a pilot cross-sectional study using baseline data collected from our pilot, assessor-blinded, parallel (1:1) randomized controlled trial (RCT) [44]. The RCT involved *n* = 21 PWD in the usual care group and *n* = 21 PWD in the exercise plus usual care group who resided in two local residential care facilities.

### 2.2. Eligibility Criteria

The inclusion criteria were met if participants (1) were aged 55 years and above; (2) lived in a residential care facility; (3) had any type of dementia confirmed by past medical history and/or a physician; (4) were able to communicate in English via reading, writing, and speaking with acceptable visual and auditory acuity; (5) were able to walk 4 m without the help of another person; (6) were able to provide informed consent via a legally authorized representative; (7) were able to provide assent; (8) were able to comprehend and follow instructions; (9) had an estimated life expectancy of ≥12 months determined by a healthcare provider; (10) were able to stand unassisted for 30 s. We excluded those (1) who were unable to comprehend or follow instructions, (2) with a severe psychiatric condition, (3) with delirium, (4) with an acute medical condition, (5) with recent surgery impairing mobility, (6) who were enrolled in another research study, (7) with severe blindness, (8) who were not able to communicate, or (9) receiving hospice care. 

### 2.3. Recruitment 

We recruited all-cause PWD from one local nursing home and one local assisted living facility. These local sites allocated space for the assessments.

### 2.4. Measures and Variables 

**Figure 1:**
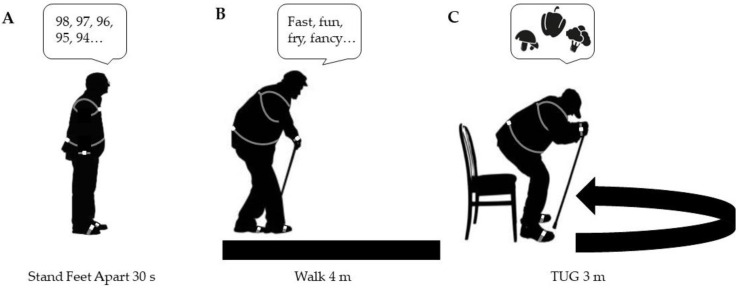
Single- and dual-task assessment schematic for (**A**) standing with feet apart for 30 s with no cognitive task and while counting backward by 1’s; (**B**) walking 4 m with no cognitive task while naming words that start with the letters F, A, or S; (**C**) timed-up-and-go (TUG) with no cognitive task and while completing a category task.

We used APDM inertial sensors (Opal v3, APDM Wearable Technologies Inc., Portland, OR, USA) to measure single- and dual-task posture, gait, and functional mobility (Figure 1). We counterbalanced the single- and dual-task assessments such that participants completed one of three random assessment sequences to account for order effects. We placed a total of six inertial sensors on the following segments: sternum, lumbar spine around L3–L4 (i.e., on the spine between the superior aspects of the iliac crests), bilateral wrists, and bilateral dorsum of each foot. APDM inertial sensors exhibited moderate to excellent validity and reliability in measuring spatiotemporal gait parameters, stability, and joint kinematics in a variety of populations [45].

### 2.5. Posture

Single- and dual-task posture involved standing with feet shoulder-width apart while completing no cognitive task, as well as while counting backward aloud by 1’s for 30 s, respectively. Participants were asked to stand with their hands down by their sides and gaze at a target at eye-level 2 m away with their eyes open. We examined sway frequency (Hz), jerk (m^2^/s^5^), mean velocity (m/s), and sway area (°/s^2^).

### 2.6. Gait

Single- and dual-task gait involved walking 4 m with no cognitive task as well as while naming as many words as possible starting with the letters F, A, or S, respectively [46]. Participants were asked to walk at their usual pace. Outcome measures included gait speed (m/s), double limb support (%), stride length (m), mid-swing elevation (cm), and medial–lateral trunk range of motion (°).

### 2.7. Functional Mobility

For single- and dual-task functional mobility, participants completed the timed-up-and-go (TUG) with no cognitive task, as well as while completing a category task, including naming fruits or animals [25,47]. The category dual-task is valid and is especially recommended for those with subtraction operation difficulties [48]. The TUG involved getting up from a chair, walking 3 m, turning around, walking back, and sitting down. Participants were asked to walk at their usual pace. Time was started on “go”, and the trial was finished once participants returned to the chair with their backs on the backrest. Outcome measures included TUG duration (s), turn angle (°), turn velocity (°/s), and sit-to-stand lean angle (°).

### 2.8. Global Cognition

The Montreal Cognitive Assessment (MoCA) measures global cognition. The MoCA also measures executive function, short-term memory recall, visuospatial abilities, attention, concentration and working memory, language, and orientation to time and place [49,50]. A MoCA score between 9–21 points indicates mild dementia, 5–8 points indicates moderate dementia, and any score of 4 points and under indicates severe dementia [51,52].

### 2.9. Descriptive Measures

The Morse Fall Scale is a 6-item measure of fall risk. The items in the scale are comprised of history of falling, secondary diagnosis, ambulatory aids, intravenous therapy, gait, and mental status. A Morse Fall Scale score of 0–24 represents no risk, 25–30 represents low risk, and ≥51 represents high risk [53].

The Functional Comorbidity Index includes 18 evenly weighted comorbidities that stratify on physical functional status. It is a better predictor of general health status than other measures of comorbidity, such as the Charleston Comorbidity Index [54]. The total number of comorbidities (0–18) was extracted from participants’ medical charts [55].

### 2.10. Data Analysis 

We conducted the analysis in SPSS version 28.0 with a significance level of α ≤ 0.05. We checked for normality and calculated descriptive statistics (means, standard deviations, percentages, and frequencies) for all variables. If Mauchly’s Test of Sphericity was significant, we used the Greenhouse–Geiser correction. We used a Bonferroni correction when examining significant effects.

In order to determine the influence of dual-tasks on postural performance by dementia severity, we conducted a repeated measures analysis of variance (RM-ANOVA) on Group (mild PWD vs. moderate-to-severe PWD) by Task (single- vs. dual-task) with repeated measures on Parameter (sway frequency, jerk, velocity, and sway area). In order to determine the influence of dual-tasks on gait performance by dementia severity, we conducted an RM-ANOVA on Group (mild PWD vs. moderate-to-severe PWD) by Task (single- vs. dual-task) with repeated measures on Parameter (gait speed, double limb support, stride length, mid-swing elevation, and trunk range of motion in the medial–lateral direction). In order to determine the influence of dual-tasks on functional mobility performance by dementia severity, we conducted an RM-ANOVA on Group (mild PWD vs. moderate-to-severe PWD) by Task (single- vs. dual-task) with repeated measures on Parameter (duration, turn angle, turn velocity, and sit-to-stand lean angle). Follow-up two-sided independent samples *t*-tests were conducted on significant Group × Task × Parameter interactions. Follow-up two-sided paired samples t-tests were conducted on significant Task × Parameter interactions.

We completed a binary logistic regression to determine whether there was a relationship between dementia severity and dual-task interference during posture, gait, and functional mobility. The degree of dual-task interference was represented by the percent of dual-task effect (DTE) and was calculated as follows [56]:

DTE(%) = (dual-task − single-task)/single-task × 100%

We separated mild PWD (MoCA range = 13 to 19 points) from moderate-to-severe PWD (MoCA range = 0 to 8 points) in this regression. Variables were removed from the model if they were correlated at *r* ≥ 0.3 and/or had a variance inflation factor of ≥2 to control for collinearity [57]. Accordingly, DTE jerk, DTE gait speed, DTE mid-swing elevation, DTE ML ROM, and DTE TUG duration variables remained in the regression analysis. Age and body mass index were treated as covariates.

## 3. Results

### 3.1. Descriptive Statistics

PWD were approximately 81 years old, and 40% were female. All participants stood independently during the postural task (*n* = 30). One participant could not walk without an assistive device and did not complete the gait or TUG tests (*n* = 29). Twelve participants used a walker, two used a cane, and eleven walked without an assistive device during the gait and TUG tests (Table 1).

### 3.2. The Influence of Dual-Tasks on Posture, Gait, and Functional Mobility by Dementia Severity

**Figure 2:**
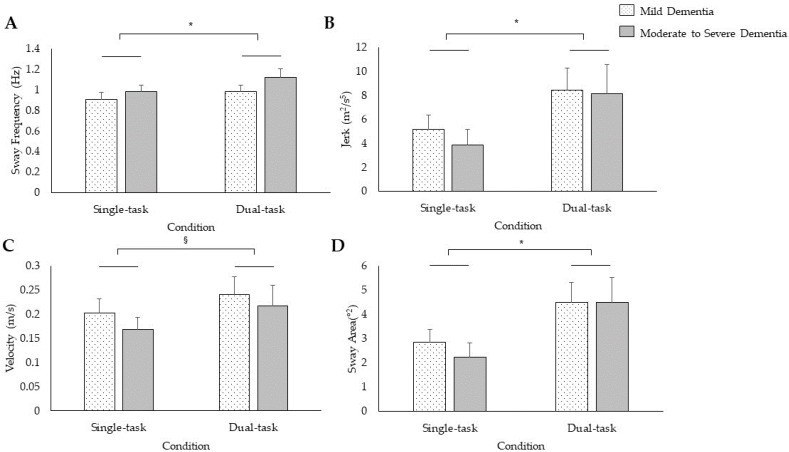
Single- and dual-task performance when standing with feet apart for 30 s for the mean and standard error of sway frequency (**A**), jerk (**B**), velocity (**C**), and sway area (**D**). * *p* < 0.05; ^§^ *p* = 0.07.

There was no Group × Task × Parameter interaction (*F*_(1.25,34.90)_ = 0.12, *p* = 0.79, *η_p_*^2 ^= 0.004) or main effect of Group (*F*_(1,28)_ = 0.15, *p* = 0.70, *η_p_*^2 ^= 0.005) for posture. There was a significant Task × Parameter interaction for posture (*F*_(1.25,34.90)_ = 5.69, *p* = 0.02, *η_p_*^2 ^= 0.17). Post hoc *t*-tests revealed that dual-task posture prompted greater sway frequency (*t*_29_ = −2.72, *p* = 0.01), jerk (*t*_29_ = −2.56, *p* = 0.02), and sway area (*t*_29_ = −3.22, *p* = 0.003) relative to single-task posture while there was a trend for lower velocity in single- compared to dual-task posture (*t*_29_ = −1.87, *p* = 0.07; Figure 2).

**Figure 3:**
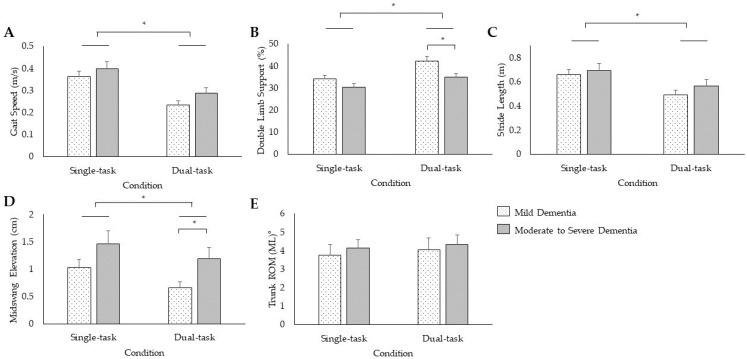
Single- and dual-task performance when walking 4 m for the mean and standard error of gait speed (**A**), double limb support (**B**), stride length (**C**), mid-swing elevation (**D**), and trunk range of motion in the medial–lateral direction (**E**). * *p* < 0.05.

There was a significant Group × Task × Parameter interaction (*F*_(1.14,30.90)_ = 6.12, *p* = 0.02, *η_p_*^2^ = 0.19) and a trend for a main effect of Group (*F*_(1,27)_ = 4.08, *p* = 0.05, *η_p_*^2^ = 0.13) for gait. Follow-up two-sided independent samples t-tests revealed that mild PWD exhibited greater double limb support percentage during dual-tasks (42.2 ± 8.3%) than moderate-to-severe PWD (35.0 ± 5.3%; *t*_27_ = −2.73, *p* < 0.01). Mild PWD also exhibited lower mid-swing elevation during dual-tasks (0.66 ± 0.43 cm) than moderate-to-severe PWD (1.2 ± 0.75 cm; *t*_27_ = 2.39, *p* < 0.02). There was a significant Task × Parameter interaction for gait (*F*_(1.14, 30.90)_ = 82.26, *p* < 0.001, *η_p_*^2^ = 0.75). Post hoc t-tests revealed that the dual-task condition elicited slower gait speed (*t*_28_ = 15.05, *p* < 0.001), greater double limb support (*t*_28_ = −8.57, *p* < 0.001), reduced mid-swing elevation (t_28_ = 5.90, *p* < 0.001), and shorter stride length (*t*_28_ = 9.44, *p* < 0.001) during dual-task gait compared to single-task gait, with no differences in single- compared to dual-task medial–lateral trunk sway (*t*_28_ = −1.27, *p* = 0.22; Figure 3).

**Figure 4:**
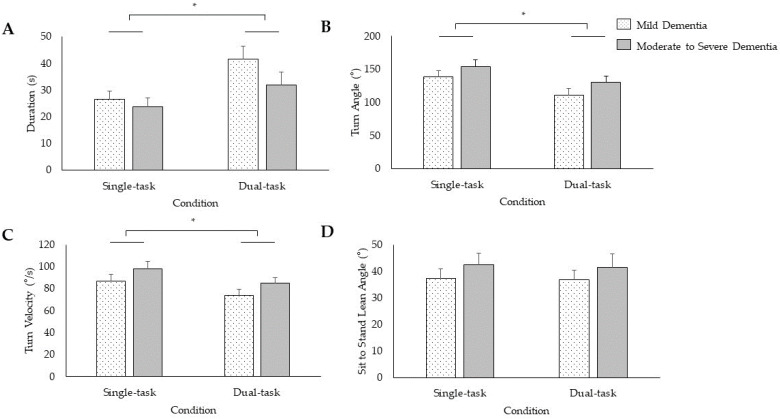
Single- and dual-task performance during the timed-up-and-go test for the mean and standard error of duration (**A**), turn angle (**B**), turn velocity (**C**), and sit-to-stand lean angle (**D**). * *p* < 0.05.

There was no Group × Task × Parameter interaction (*F*_(1.47,39.80)_ = 0.51, *p* = 0.55, *η_p_*^2 ^= 0.02) or main effect of Group (*F*_(1,27)_ = 2.15, *p* = 0.15, *η_p_*^2 ^= 0.07) for functional mobility. There was a significant Task × Parameter interaction for functional mobility (*F*_(1.47,39.80)_ = 30.32, *p* < 0.001, *η_p_*^2 ^= 0.53). The dual-task TUG resulted in a longer time to completion (*t*_28_ = −6.15, *p* < 0.001), reduced turn angle (*t*_28_ = 5.14, *p* < 0.001), and slower turn velocity (*t*_28_ = 5.89, *p* < 0.001) relative to the single-task TUG, with no differences in single- compared to dual-task lean angle (*t*_28_ = 0.48, *p* = 0.63; Figure 4).

### 3.3. Relationship between Dementia Severity and Dual-Task Interference

The overall logistic regression model for dementia severity and dual-task interference during posture, gait, and functional mobility was statistically significant (*X^2^*_(7)_ = 27.75, Cox and Snell *R*^2^ = 0.62, *p* < 0.001; Table 2). There was a trend for an association between dementia severity and the dual-task interference effect for jerk, such that smoother dynamic stability during the dual-task condition was related to mild PWD (85.4% increase in jerk from single- to dual-tasks) compared to moderate-to-severe PWD (324.2% increase in jerk from single- to dual-tasks; *β *= −0.01, *p* = 0.08). There was a significant association between dementia severity and the dual-task interference effect for gait speed, such that slower gait speed during the dual-task was correlated to mild PWD (−35.7% decrease in gait speed from single- to dual-tasks) compared to moderate-to-severe PWD (−27.6% decrease in gait speed from single- to dual-tasks; *β* = −0.24, *p* = 0.046). All the other DTE parameters for posture, gait, and functional mobility were not significant (*p* > 0.05).

## 4. Discussion

### 4.1. Main Findings

In the present study, dual-tasks resulted in greater instability, cautious gait, and impaired functional mobility compared to single-tasks. To the best of our knowledge, this is the first study that has examined whether dual-tasks are impacted across dementia severities. Notably, dual-task performance and interference were related to dementia severity. Specifically, dual-task performance was impacted (reduced double limb support, greater mid-swing elevation), and dual-task interference (greater jerk, faster gait speed) was related to moderate-to-severe compared to mild PWD.

### 4.2. The Influence of Dual-Tasks on Posture, Gait, and Functional Mobility in PWD

Our study corroborates previous work exhibiting greater sway area and path length during the dual-task compared to single-task condition in community-dwelling PWD [15,34] and adds that sway frequency and jerk were also increased in our sample living in residential care. Greater sway frequency during quiet standing, particularly between 1.0 and 5.0 Hz, is characteristic of trunk and leg segment antiphase oscillations [58]. The greater sway frequency observed in dual-tasks may be suggestive of both impairments in neural control as well as uncoordinated postural control patterns [59]. Additionally, greater jerk is indicative of poorer dynamic stability [60]. Recent work has linked cortical thinning with lower cognitive–motor automaticity among PWD [61], which may partly explain this dual-task instability.

Dual-tasks promoted a unique gait signature relative to single-tasks among PWD. Specifically, participants adopted a cautious gait pattern during dual-task gait by exhibiting slower gait speed, greater double limb support, and shorter stride length when compared to single-task gait. Similarly, compared to single-task functional mobility, the dual-task functional mobility assessment exposed prolonged time to complete the TUG, reduced turn angle, and slower turn velocity, indicating greater instability. Participants likely adopted a cautious gait strategy in response to greater attention demand [62] in an effort to allocate attention resources to both tasks [63]. However, the lower mid-swing elevation during the dual-task was a maladaptive gait strategy that increased the risk of tripping. This altered gait pattern was likely a consequence of a central misprocessing of information that includes attention and executive functions [36,41,64]. As a result, dual-tasks provoked PWD to adopt an unstable gait and functional mobility pattern. Our findings highlight that dual-task assessment may be sensitive to detect further impairments in posture, gait, and functional mobility compared to single-task assessment.

### 4.3. The Influence of Dementia Severity on Dual-Task Performance and Dual-Task Interference

Previous work has revealed that single-task balance and gait control progressively deteriorate from mild to moderate dementia [65]. Our study contributes new knowledge that dual-task performance and interference may be impacted by dementia severity. For standing posture, we observed a trend for less dual-task interference for jerk in mild PWD (85.4% increase in jerk from single- to dual-tasks) compared to moderate-to-severe PWD (324.2% increase in jerk from single- to dual-tasks). Thus, it appears that greater dementia severity may impair the ability to control smooth postural motion. We postulate that dual-tasks may generate a greater relative impact on dynamic stability than single-tasks for individuals with greater cognitive decline.

For gait, mild PWD exhibited greater double limb support and lower mid-swing elevation during dual-tasks relative to moderate-to-severe PWD, which extends previous work exhibiting single-task gait impairments by dementia severity [65]. Mild PWD also experienced greater dual-task interference for gait speed (−35.7% decrease in gait speed from single- to dual-tasks) than moderate-to-severe PWD (−27.6% decrease in gait speed from single- to dual-tasks). Greater double limb support and lower mid-swing elevation seem to coexist with slower gait speeds [66]. Our findings suggest that mild PWD employed a cautious gait strategy during dual-tasks by appropriately increasing double limb support and reducing gait speed to a greater degree than moderate-to-severe PWD in an effort to maintain stability. Impulsivity and risk-taking have been observed in PWD [67]. Older adults who fall often attribute their accidents to hurrying and missing important cues in the environment [68]. Additionally, wandering is more common in later stages of dementia and has been shown to impact physical activity levels [69]. Our results convey that those with further cognitive decline may have a reduced decision-making ability to select an appropriate gait strategy during dual-tasks, inadvertently rush, and increase their risk for falls. It is also possible the other symptoms of greater dementia severity, such as wandering [69], may contribute to altered gait patterns. It is important to note that all of our participants had slow single- and dual-task gait, as defined by a threshold of <0.8 m/s, which is indicative of low functional ability [20]. Thus, solely employing a slow gait threshold would have been insufficient to identify individuals across the dementia continuum. The lack of a relationship between measures of functional mobility and dementia severity may posit that dual-task functional mobility measures neural control processes that are independent of, and in addition to, the traditional, clinical signs of dementia, but further work is needed to confirm these findings. Taken together, dual-task performance for double limb support and mid-swing elevation, as well as dual-task interference for jerk and gait speed may be sensitive biomarkers to track dementia progression from mild dementia to moderate-to-severe dementia. As such, we speculate that PWD with greater cognitive decline are at a higher risk for falls due to impairments in dual-tasks.

### 4.4. Implications

Clinicians can use wearable inertial sensors to objectively measure dual-task posture, gait, and functional mobility, as the sensors are portable, simple to use, quick to administer, have a clear interpretation of the measurement results, and are relatively inexpensive. Our results suggest that PWD are at a greater risk for mobility impairments in their daily lives during dual-tasks compared to single-tasks. Dual-tasks were more sensitive to detect impairments in posture, gait, and functional mobility than single-tasks, and should be considered for routine risk assessments among PWD. Dual-task performance in double limb support and mid-swing elevation, as well as interference in jerk and gait speed, seem to be promising markers that may distinguish between mild compared to moderate-to-severe PWD, signifying that they could be considered as cognitive screening and monitoring tools. Given that moderate-to-severe PWD have poorer dual-task performance for double limb support and mid-swing elevation, as well as greater dual-task interference for jerk and gait speed than mild PWD, targeting the improvement of dual-tasks should be considered in therapeutic interventions in this population. For those in low- and middle-income countries with no access to wearable inertial sensors, clinicians should target the improvement of dual-tasks among PWD and use a stopwatch to measure dual-task gait speed.

### 4.5. Limitations

These findings are only generalizable to PWD in residential care facilities who are able to stand and walk without the assistance of another person. We only recruited from two residential care facilities, and participants had varying functional impairments. Most participants took several medications. Our participant sample included only 40% females, even though females are more likely to acquire dementia, and it is unclear whether this affected the results [1]. The Morse Fall Scale is less sensitive to predicting falls compared to other fall assessments [70]. Our small sample size limited our RM-ANOVA and regression analyses. Future research in larger samples should consider determining whether dual-task performance and interference are sensitive to distinguish between mild, moderate, and severe PWD. It was not possible to determine whether participants were prioritizing their attention on the motor or cognitive task during the dual-task conditions. While all participants had a confirmed diagnosis of dementia in their medical charts, the dementia subtype was often not recorded; this may limit our findings in terms of the root cause of dementia and potential dementia subtype manifestations. Larger and longitudinal studies of postural sway, gait, and functional mobility are needed to determine if acceleration parameters are sensitive descriptors of dementia progression and, hence, are useful in clinical trials of neuroprotective interventions.

## 5. Conclusions

Dual-task jerk, double limb support, lower mid-swing elevation, and gait speed were more sensitive to detect impairments in posture, gait, and functional mobility than single-task assessments among PWD in residential care facilities. Mild PWD had better dynamic stability and a superior ability to appropriately select a cautious gait strategy by increasing double limb support and reducing gait speed during dual-tasks than moderate-to-severe PWD. Our work suggests that dual-tasks should be considered in routine assessments to monitor cognitive decline among PWD and identify those with increased impairment in posture, gait, and functional mobility in residential care facilities.

## Figures and Tables

**Table 1:** Participant characteristics for PWD; mean ± standard deviation (range).

Variable	All Participants (*n* = 30)	Mild Dementia (*n* = 16)	Moderate-to-Severe Dementia (*n* = 14)
Age, years	81.3 ± 7.1 (68–98)	81.0 ± 8.5 (68–98)	81.6 ± 5.5 (69.0–91.0)
Female Sex, n (%)	12 (40.0)	8 (26.7)	4 (13.3)
BMI, kg/m^2^	21.5 ± 4.1 (13.5–33.4)	23.0 ± 4.3 (17.0–33.4)	19.9 ± 3.4 (13.5–29.3)
FCI, total number	3.4 ± 2.0 (1–8)	4.5 ± 1.9 (2–8)	2.1 ± 1.4 (1–6)
Medications, total number	12.3 ± 5.9 (0–28)	12.1 ± 4.3 (7–24)	12.6 ± 7.6 (0–28)
Morse Fall Scale, points	38.2 ± 22.0 (15–80)	48.8 ± 22.5 (15–80)	26.1 ± 14.2 (15–55)
MoCA, points	10.4 ± 6.0 (0–19)	15.6 ± 2.1 (13–19)	4.6 ± 2.6 (0–8)
Dementia Type, n (%)			
Alzheimer’s Disease	9 (30.0)	4 (25.0)	5 (35.7)
Vascular Dementia	2 (6.7)	2 (12.5)	0
Parkinson’s Disease Dementia	1 (3.3)	1 (6.3)	0
Alcohol-Induced Dementia	1 (3.3)	1 (6.3)	0
Unspecified, Without Behavioral Disturbance	17 (56.7)	8 (50.0)	9 (64.3)
Dementia Onset, years	2.8 ± 2.8 (0.3 to 9.3)	2.9 ± 2.7 (0.4 to 9.3)	2.4 ± 3.6 (0.3 to 6.6)
Site			
Nursing Home, n (%)	15	14	1
Assisted Living, n (%)	15	2	13
Mobility Device, n (%)			
Wheelchair	1 (3.3)	1	
Walker	15 (50.0)	12	3
Cane	3 (10.0)	1	2
None	11 (36.7)	2	9

BMI—body mass index; FCI—Functional Comorbidity Index; MoCA—Montreal Cognitive Assessment.

**Table 2:** Association between dementia severity and dual-task effect (DTE) for posture, gait, and functional mobility among PWD (*n* = 29).

Outcome	β	SE	Wald	*p*-value	OR	95% CI Lower	Upper
Constant	−37.75	31.29	1.46	0.23	<0.001	---	---
Age	0.05	0.21	0.05	0.82	1.05	0.69	1.60
BMI	1.05	0.66	2.54	0.11	2.89	0.79	10.39
DTE jerk ˆ	−0.01	0.01	3.05	0.08 ^§^	0.99	0.98	1.00
DTE gait speed ^†^	−0.24	0.12	3.99	0.046 *	0.79	0.62	0.996
DTE mid-swing elevation	−0.03	0.05	0.40	0.53	0.97	0.89	1.06
DTE ML ROM	−0.001	0.05	<0.001	0.99	1.00	0.91	1.10
DTE TUG duration	0.11	0.07	2.46	0.12	1.11	0.97	1.27

*X*^2^_(7)_ = 27.75, Cox and Snell *R*^2^ = 0.62, *p* < 0.001, 93.3% prediction accuracy for mild PWD; 92.9% prediction accuracy for moderate-to-severe PWD. Covariates included Age and BMI: body mass index. Dementia severity was defined as 1: Montreal Cognitive Assessment = 0 to 10 (*n* = 14) and 2: Montreal Cognitive Assessment = 11–19 (*n* = 15); ML ROM: medial–lateral range of motion during gait; TUG—timed-up-and-go. SE—standard error; OR—odds ratio; CI—confidence interval; BMI—body mass index; ML ROM—medial–lateral range of motion; TUG—timed-up-and-go. ˆ β indicative of a trend for smoother dynamic stability during dual-tasks in mild PWD than in moderate-to-severe PWD. ^† ^β indicative of greater dual-task interference (i.e., cautious slow gait dual-task strategy) in mild PWD than in moderate-to-severe PWD. * *p* < 0.05; ^§^ *p* = 0.084.

## Data Availability

The data presented in this study are available upon request from the corresponding author.
